# Rejoinder of Dr. Wm. Stokes, of Baltimore, to an Editorial Article in the American Journal of Insanity, Containing Strictures upon the Mount Hope Institution

**Published:** 1848-11

**Authors:** 


ART. V.—*Rejoinder of Dr. Wm. H. Stokes, of Baltimore, to an editorial article in the American Journal of Insanity, containing Strictures upon the Mount Hope Institution.*

We give insertion to the letters of Dr. Stokes, recognising the right of a respectable member of the Profession to be heard through the columns of any public journal he may select, under such circumstances, provided the rules of proper courtesy are observed. In so doing (it is probably needless to state) our journal is only a vehicle of the communication, the editor being in nowise committed on behalf of the respondent. Of the merits of the controversy we are not, of course, competent to judge, but must presume that the strictures of which Dr. S. complains, if uncalled for, were made under misapprehensions. EDITOR.

AUSTIN FLINT, M. D., BALTIMORE, Oct. 3. 1848.
*Editor of Buffalo Medical Journal.*

DEAR SIR:—I beg leave to send you the following short rejoinder to the strictures contained in the last number of the American Journal of Insanity, and written by Dr. Brigham, on the subject of Mount Hope Institution. If you think proper to give it an insertion in your excellent Journal, I shall feel obliged to you. I have the honor to remain,

Very respectfully,
Your Ob't. Servant,
WM. H. STOKES.

Dr. BRIGHAM,
*Editor of the Journal of Insanity.*

SIR:—I must be allowed to notice briefly the remarks contained in the last number of the Journal of Insanity, and written by you, in regard to Mount Hope Institution. I must be permitted to express my deep regret, hat you should have inspected the institution under the influence of feelings, which prevented your forming an unbiassed and correct judgment of its arrangements and medical supervision. Otherwise, I am sure, you would not have given expression to statements so prejudicial to the establishment, and yet so wholly at variance with fact. You have not only expressed opinions well calculated to injure it without the shadow of evidence, but you have asserted, in regard to myself, as its Physician, what is perfectly destitute of foundation.

As regards our means of heating and ventilation, you have not the slightest ground for saying they are defective. The arrangements for warmth and ventilation are unsurpassed by any similar establishment in this Country or in Europe. I say this advisedly, having myself visited most of any repute. The new wing and centre are heated by hot air furnaces in the basement. These supply the apartments and hall with a constant and regular influx of warm pure air, and in such abundance as to maintain the whole at a pleasant uniform temperature in the coldest weather in winter. The old wing and the Lodge are equally well heated by well-arranged and well protected stoves. In reference to our means of *ventilation*, it is invariably remarked by visiters, with surprise, that there should exist here such a perfect freedom from the closeness and impurity of air observed, almost without exception, in other establishments. Most of our sleeping apartments for single patients are considerably larger than those of other asylums. With us they average 9 by 14 feet, with a ceiling of 11 feet in height. In most other asylums they are but 9 by 10 feet, and 9 feet high. To afford the most ample circulation of air throughout the room, we have also over each room an unglazed transom sash, one foot four inches high. The air is consequently always pure and healthy.

You remark further, that we are defective in facilities for affording labor to patients. Perhaps we are unfortunate in not having a class of patients similar to that found in State Institutions for the Insane—a class who have all their lives been accustomed to hard labor, and who therefore manifest no great reluctance to engage in this kind of employment when attacked with mental derangement and confined in an asylum. You forget that this is a private establishment, and resorted to by patients of a wholly different class—patients who *may be amused* and perhaps induced to engage *in light occupation*, but who *will not work*. At no period of their lives have they been habituated to manual labor, and it would require more coercion than we are willing to subject our patients to, to induce them to labor now. But as it respects the possession of the most ample and abundant means for affording agreeable occupation and amusement to each and every patient, I do know, that this institution is behind none other in the country.

You have also ventured to pass an opinion condemnatory of the *medical supervision* of this institution. You assert that "he (Dr. Stokes) is, we understand, only hired to visit the institution daily and prescribe for such patients as the Sisters request." This statement is perfectly gratuitous and destitute of the least foundation. I make my visits at the institution morning and evening, and more frequently if necessary. Every patient in the house is under my medical charge, and is seen and prescribed for by me. During the six years that I have presided over it in the capacity of the Physician, there has been no exception to this regulation, and it is not true that I prescribe for such patients only as the Sisters request.

Contrary to your belief and expectation, I may be allowed to assure you, that I have experienced no difficulty whatever in establishing a uniform and good *system* of moral treatment, and that too, " without having entirely the selection, instruction and discharge of the attendants on the patients entirely in my own hands." The patients of Mount Hope are happy in possessing the priceless services of the Sisters of Charity. They constitute here the corps of attendants, and I presume you would hardly expect or desire the Physician to possess over them an appointing and expelling power. They are possessed of a degree of refinement and intelligence infinitely above the ordinary class of attendants, and impelled, as they are, to the performance of their duties by the highest and holiest motives that can influence human action, I declare to you, that I experience *no difficulty in establishing, with their co-operation, a uniform and good system of moral treatment.*

But, sir, if you would wish to form an accurate judgment of the adaptation of our arrangements to the exalted object of restoring the diseased mind, and of the sufficiency of the *medical supervision,* I refer you with pride to the large per-centage of recoveries recorded in the different reports for the last three years. Examined by this test, we do not fear comparison with any other institution. This large proportion of recoveries will amply testify to the completeness of our arrangements, and their perfect adaptation to the high purpose of restoring the disordered mind to its healthy balance. Whatever may be your opinion of the subject, formed from a hurried inspection of the building, experience has demonstrated, in the most satisfactory manner, that the institution is wanting in nothing that can contribute to the recovery, or promote the comfort of our patients.

Your Obedient Servant,

Wm. H. Stokes.

*Physician to Mount Hope.*

